# Urokinase-type plasminogen activator and arthritis progression: role in systemic disease with immune complex involvement

**DOI:** 10.1186/ar2946

**Published:** 2010-03-02

**Authors:** Andrew D Cook, Christine M De Nardo, Emma L Braine, Amanda L Turner, Ross Vlahos, Kerrie J Way, S Kaye Beckman, Jason C Lenzo, John A Hamilton

**Affiliations:** 1 Arthritis and Inflammation Research Centre, Department of Medicine, The University of Melbourne, Parkville, Melbourne, Victoria 3010, Australia; 2 Cooperative Research Centre for Chronic Inflammatory Diseases, The University of Melbourne, Parkville, Melbourne, Victoria 3010, Australia

## Abstract

**Introduction:**

Urokinase-type plasminogen activator (u-PA) has been implicated in fibrinolysis, cell migration, latent cytokine activation, cell activation, T-cell activation, and tissue remodeling, all of which are involved in the development of rheumatoid arthritis. Previously, u-PA has been reported to play a protective role in monoarticular arthritis models involving mBSA as the antigen, but a deleterious role in the systemic polyarticular collagen-induced arthritis (CIA) model. The aim of the current study is to determine how u-PA might be acting in systemic arthritis models.

**Methods:**

The CIA model and bone marrow chimeras were used to determine the cellular source of u-PA required for the arthritis development. Gene expression of inflammatory and destructive mediators was measured in joint tissue by quantitiative PCR and protein levels by ELISA. The requirement for u-PA in the type II collagen mAb-induced arthritis (CAIA) and K/BxN serum transfer arthritis models was determined using u-PA^-/- ^mice. Neutrophilia was induced in the peritoneal cavity using either ovalbumin/anti-ovalbumin or the complement component C5a.

**Results:**

u-PA from a bone marrow-derived cell was required for the full development of CIA. The disease in u-PA^-/- ^mice reconstituted with bone marrrow from C57BL/6 mice was indistinguishable from that in C57BL/6 mice, in terms of clincal score, histologic features, and protein and gene expression of key mediators. u-PA^-/- ^mice were resistant to both CAIA and K/BxN serum transfer arthritis development. u-PA^-/- ^mice developed a reduced neutrophilia and chemokine production in the peritoneal cavity following ovalbumin/anti-ovalbumin injection; in contrast, the peritoneal neutrophilia in response to C5a was u-PA independent.

**Conclusions:**

u-PA is required for the full development of systemic arthritis models involving immune complex formation and deposition. The cellular source of u-PA required for CIA is bone marrow derived and likely to be of myeloid origin. For immune complex-mediated peritonitis, and perhaps some other inflammatory responses, it is suggested that the u-PA involvement may be upstream of C5a signaling.

## Introduction

Urokinase-type plasminogen activator (u-PA) is a serine protease that, along with tissue-type plasminogen activator (t-PA), cleaves plasminogen to form plasmin [1]. The plasminogen activator (PA)/plasmin system has been implicated in the following processes in both physiology and pathology: fibrinolysis, cell migration, latent cytokine activation, cell activation via u-PA receptor (u-PAR), T-cell activation, and tissue remodeling (directly or indirectly via matrix metalloprotease (MMP) activation) (reviewed in [1,2]).

Rheumatoid arthritis is a chronic systemic inflammatory disease of unknown etiology, characterized by synovial hyerplasia, infiltration of inflammatory cells, intra-articular fibrin deposition and erosion of cartilage and bone. Enhanced u-PA and reduced t-PA activity in the synovium have been associated with rheumatoid arthritis severity [3]. Increased levels of u-PAR, and the PA inhibitors PAI-1 and PAI-2, are also found in rheumatoid arthritis tissue [3]. Several different cell types present in arthritic joints can produce PAs and their inhibitors *in vitro*, including in response to inflammatory cytokines [4-11]. We and other workers have previously reported that u-PA plays a protective role in the antigen-induced arthritis (AIA) model [12] and the mBSA/IL-1 monoarticular arthritis model [13], with u-PA gene-deficient (u-PA^-/-^) mice developing more severe disease associated with increased intra-articular fibrin deposition. In the chronic systemic collagen-induced arthritis (CIA) model, however, we [14] and other workers [15] have found that u-PA was deleterious, with u-PA^-/- ^mice developing very mild disease and little fibrin deposition. In addition, the T-cell proliferative response to type II collagen (CII) was reduced in u-PA^-/- ^mice, although the antibody response to CII was normal [14]. Information on why u-PA depletion has differing outcomes in these various arthritis models is lacking but is essential if u-PA targeting is to be considered as a therapeutic strategy in rheumatoid arthritis and other inflammatory conditions.

In order to examine how u-PA might be acting in the systemic arthritis models, the following parameters were assessed: the cellular source of u-PA required for CIA development; inflammatory and destructive mediator expression in the joints of C57BL/6 mice and u-PA^-/- ^mice following CIA development; the requirement for u-PA in the development of the CII mAb-induced arthritis model (CAIA) and the K/BxN serum transfer model of arthritis, both of which do not require B cells or T cells, at least for the initiation of disease [16-18]; and the requirement for u-PA in the development of immune complex-mediated neutrophilia in the peritoneal cavity. We show that u-PA produced by a bone marrow-derived cell is important for the full development of CIA; many inflammatory and destructive mediators are increased in the joints of C57BL/6 mice but not of u-PA^-/- ^mice following CIA development; u-PA^-/- ^mice are essentially resistant to both CAIA and the K/BxN serum transfer model of arthritis; and immune complex-mediated neutrophilia and chemokine production in the peritoneal cavity is u-PA dependent, whereas C5a-mediated neutrophilia is not, suggesting u-PA is acting upstream of C5a signaling.

## Materials and methods

### Mice

The u-PA^-/- ^mice, provided by Dr P Carmeliet (University of Leuven, Belgium), were backcrossed onto the C57BL/6 background for 11 generations. C57BL/6 CD45 congenic mice, expressing the Ly5.1 allotype, were obtained from Walter and Eliza Hall Institute Animal Supplies (Parkville, Victoria, Australia). All strains were bred in our onsite animal facility, fed standard rodent chow and water ad libitum, and housed in sawdust-lined cages in groups of five. Mice of both sexes, 8 to 12 weeks of age, were used in all experiments. All experiments were approved by The Royal Melbourne Hospital Research Foundation Animal Ethics Committee.

### Bone marrow transplantation

The u-PA^-/- ^or C57BL/6 recipient mice received total body irradiation (two exposures × 5.5 Gy, 3 hours apart). Bone marrow cells were harvested from the femurs and tibiae of C57BL/6 or u-PA^-/- ^donor mice expressing the Ly5.1 allotype of CD45. Recipient mice (expressing the Ly5.2 allotype of CD45) were injected intravenously with 5 × 10^6 ^bone marrow cells. Effective bone marrow reconstitution was determined by flow cytometry analysis of peripheral blood leukocytes 6 weeks later using the different congenic CD45 allotypes (Ly5.1 and Ly5.2). Six weeks after irradiation, 95 ± 1% of circulating leukocytes expressed the phenotypic marker of the donor bone marrow.

### Collagen-induced arthritis

Mice were immunized intradermally in the base of the tail with 100 μg chick CII (Sigma, St Louis, MO, USA), emulsified in an equal volume of complete Freund's adjuvant containing 5 mg/ml heat-killed *Mycobacterium tuberculosis *(H37 Ra; Difco, Detroit, MI, USA). This procedure was repeated as a boost 21 days later, as previously published [14].

Animals were assessed for redness and swelling of limbs and a clinical score was allocated for each limb using an established scoring system with slight modifications [14] as follows: 0 = normal; 1 = slight swelling and/or erythema; 2 = extensive swelling and/or erythema; 3 = severe swelling; 4 = rigidity. Severity of arthritis is expressed in terms of the mean clinical score (range 0 to 16 per mouse).

### Type II collagen mAb-induced arthritis model

The anti-CII mAb-producing hybridomas for M2139 and CIIC1 were a gift from Prof. R Holmdahl (Karolinska Institute, Stockholm, Sweden). The cocktail of M2139 and CIIC1 mAbs was prepared by mixing equal concentrations of each of the antibody, and mice were then injected intravenously with 4.5 mg mAb cocktail on days 0 and 1. On day 5, mice received intraperitoneally 50 μg lipopolysaccharide (*Escherichia coli *serotype 0127:B8; Sigma-Aldrich), 5 μg Pam-3-Cys (EMC Microcollections, Tübingen, Germany), or PBS. Mice were scored daily, using the same scoring system as for the CIA model.

### K/BxN serum transfer model of arthritis

K/BxN mice were bred as described previously [19]. Serum was collected up to 12 weeks of age and stored at -80°C. Serum (50 μl in 150 μl PBS) was injected intraperitoneally on days 0 and 2. Mice were scored daily, using the same scoring system as for the CIA model.

### Immune complex-mediated neutrophilia

Fifty microliters of chicken egg albumin (ovalbumin, 20 mg/kg body weight; Sigma) was injected intravenously followed by intraperitoneal injection of 1 ml rabbit polyclonal IgG rich in antibody to chicken egg albumin (anti-ovalbumin, 800 μg/mouse; Sigma), as previously described [20]. Cells were harvested 4 hours later by lavage with 5 ml ice-cold, sterile PBS. Total and differential cell counts (Diff-Quik; Lab Aids, Narrabeen, NSW, Australia) were performed on the peritoneal exudate cells [21,22].

In certain experiments, C5a (1.25 μg; HyCult Biotechnology, Uden, The Netherlands) was given intraperitoneally alone or administered together with the anti-ovalbumin, as above. In these experiments, cells were harvested 2 hours later.

### Histology

At termination following arthritis induction, the rear limbs and ankles were removed, fixed, decalcified, and paraffin embedded, as previously described [14]. Frontal sections (5 μm) were stained with either H & E to examine joint architecture or with safranin O, fast green and hematoxylin for proteoglycan loss, and were evaluated without knowledge of the experimental groups, using the histologic assessment as published [14]. Briefly, infiltration of cells, cartilage damage and bone erosions were all scored separately from 0 (normal) to 3 (severe), and proteoglycan loss was scored from 0 (normal) to 3 (complete loss of staining). These scores were added to give an overall histologic score out of 12.

### Detection of fibrin(ogen) by immunohistochemistry

Fibrin(ogen) deposition was identified in rear limbs as before [14]. Briefly, paraffin-embedded sections were deparaffinized, incubated with 1% (w/v) BSA and 5% (w/v) skim milk powder for 1 hour, and then stained with a goat anti-mouse fibrinogen/fibrin antibody (Accurate Chemical & Scientific, Westbury, NY, USA) overnight at 4°C. Endogenous peroxidase activity was blocked with 0.3% (v/v) H_2_O_2 _(Sigma) in methanol. Following washing, sections were incubated with a biotinylated donkey anti-goat IgG (Jackson ImmunoResearch, West Grove, PA, USA), followed by a streptavidin-peroxidase conjugate (BD Pharmingen, San Diego, CA, USA). Peroxidase activity was demonstrated by incubation with 3-amino-9-ethylcarbazole (Sigma). Sections were counterstained with hematoxylin.

### Preparation of joint tissue washouts

Following sacrifice, the tendons and synovium from the ankle joints of the hind limbs were dissected free from the surrounding tissue and washed in 200 μl DMEM, supplemented with HEPES (20 mM), L-glutamine (2 mM), and penicillin (50 U/ml)/streptomycin (50 μg/ml), and were incubated for 1 hour at room temperature to allow the elution of cytokines [14]. Supernatants were then removed and stored at -20°C until assayed.

### Cytokine ELISAs

TNFα and IL-1β levels were measured in ankle joint tissue washouts by ELISA (OptEIA ELISA kits; BD Pharmingen), as outlined previously [14]. TNFα and IL-1β ELISAs were sensitive down to 5 and 3 pg/ml, respectively. Keratinocyte-derived chemokine (KC) and macrophage inflammatory protein-2 (MIP-2) levels were measured in the peritoneal exudate fluid by ELISA (DuoSet; R&D Systems, Minneapolis, MN, USA), according to the manufacturer's instructions: KC and MIP-2 ELISAs were sensitive down to 2 pg/ml.

### MMP-9 expression in joint tissue washouts

Zymography was used to assess protease expression in joint tissue washouts [23]. Briefly, joint washouts from mice with the same arthritic score were pooled and concentrated. SDS-PAGE mini-gels (10%) were prepared with the incorporation of gelatin (2 mg/ml; Labchem, Pittsburgh, PA, USA) before casting. The joint washouts (20 μl) were run into gels at a constant voltage of 200 V under nonreducing conditions. When the dye front reached the bottom, gels were removed and washed twice for 15 minutes in 2.5% Triton X-100 and incubated at 37°C overnight in zymography buffer (50 mM Tris-HCl (pH 7.5), 5 mM CaCl_2_, 1 mM ZnCl_2 _and 0.01% NaN_3_). The gels were then stained for 45 minutes with Coomassie Brilliant Blue R-250 (Sigma) and extensively destained. Following destaining, zones of enzyme activity appeared clear against the Coomassie Blue background.

### Quantitative PCR analysis of gene expression

Quantitative PCR was performed as before [24]. Briefly, joints were crushed, RNA was extracted using the RNeasy Mini Kit (Qiagen, Valencia, CA, USA) and cDNA was prepared. Quantitative PCR was performed using Predeveloped TaqMan gene expression assays for TNFα, IL-1β, IL-6, MCP-1, t-PA, u-PA, u-PAR, MMP-3, MMP-9, MMP-13, ADAMTS-4 and ADAMTS-5 (Applied Biosystems, Foster City, CA, USA), and was read on an ABI Prism 7900H sequence detection system, followed by analysis using ABI Prism SDS 2.1 software. The TATA-binding protein (GeneWorks, Thebarton, SA, Australia) was used as the control gene. The comparative threshold method for relative quantification was used, and results are expressed as relative gene expression for each target gene.

### Statistical analysis

For clinical and histologic scores and cytokine levels, the Mann-Whitney two-sample rank test was used to determine the level of significance between two experimental groups. For peritonitis and gene expression studies, an unpaired Student's *t *test was used; values are expressed as the mean ± standard error of the mean. *P *≤ 0.05 was considered statistically significant.

## Results

### Bone marrow cell-derived u-PA is required for full induction of collagen-induced arthritis

Based on *in vitro *studies, several different cell types present in arthritic joints have been proposed to be potential sources of u-PA [10]. To determine the cellular source of u-PA important for the development of arthritis, bone marrow chimera experiments were performed followed by the induction of CIA.

#### u-PA production by bone marrow-derived cell(s) is sufficient

**Figure 1:**
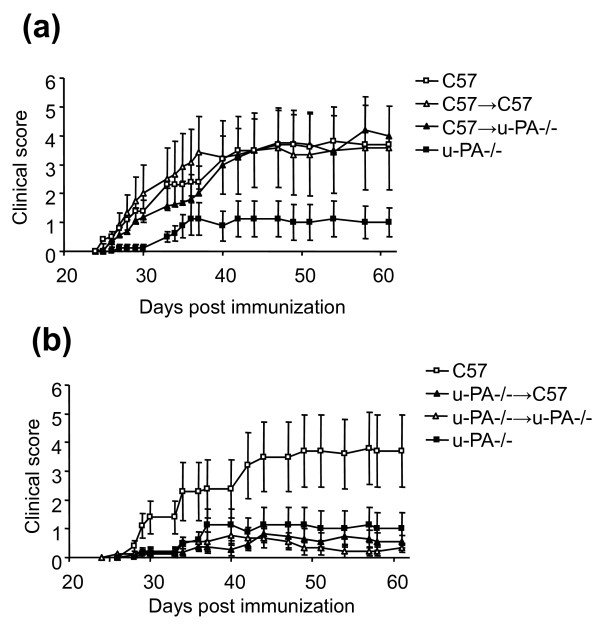
**Collagen-induced arthritis development in C57BL/6 and u-PA^-/- ^chimeric mice**. Urokinase-type plasminogen activator (u-PA) production by bone marrow-derived cells is required for full expression of collagen-induced arthritis (CIA). **(a) **Severity (mean clinical score for all mice ± standard error of the mean) for C57 (n = 10), C57 → C57 (n = 12), C57 → u-PA^-/- ^(n = 16) and u-PA^-/- ^(n = 8) mice. *P *< 0.05, C57 → u-PA^-/- ^vs. u-PA^-/- ^mice. **(b) **Severity for C57 (n = 10), u-PA^-/- ^→ C57 (n = 11), u-PA^-/- ^→u-PA^-/- ^(n = 9), u-PA^-/- ^(n = 8) mice. *P *< 0.01, u-PA^-/- ^→ C57 vs. C57 mice.

C57BL/6 mice developed CIA with an incidence of 80% (eight out of 10 mice) and average maximum clinical severity of 4.1 ± 1.2. C57BL/6 sham chimeras (C57 → C57) developed disease of a similar incidence (92%, 11 out of 12 mice) and severity (Figure 1a), with an average clinical severity of 4.4 ± 1.4, indicating that bone marrow transplantation *per se *did not affect the disease development. u-PA^-/- ^mice developed mild CIA (Figure 1a), with a cumulative incidence of 75% (six out of eight mice) and mean severity of 1.5 ± 0.6 (*P *< 0.05 compared with C57BL/6 mice), as previously reported [14]. Adoptively transferring bone marrow cells from C57BL/6 mice to irradiated u-PA^-/- ^mice (C57 → u-PA^-/-^) led to the development of CIA that was indistinguishable from that induced in C57BL/6 mice (Figure 1a), with a cumulative incidence of 69% (11 out of 16 mice) and a mean maximum clinical score of 4.9 ± 1.2. The severity of arthritis was significantly greater in these C57BL/6 → u-PA^-/- ^chimeras compared with u-PA^-/- ^mice (*P *< 0.05). u-PA produced by a bone marrow-derived cell(s) is thus sufficient for the full induction of CIA.

#### u-PA production by non-bone marrow-derived cell(s) is not sufficient

To determine whether u-PA derived from a non-bone marrow cell(s) could also restore disease in u-PA^-/- ^mice, the reverse chimera experiment was performed whereby bone marrow cells from u-PA^-/- ^mice were transferred to irradiated C57BL/6 mice (u-PA^-/- ^→ C57). u-PA^-/- ^sham chimeras, in which u-PA^-/- ^bone marrow was transferred to irradiated u-PA^-/- ^mice (u-PA^-/- ^→ u-PA), developed disease with a similar severity (Figure 1b) and incidence as u-PA^-/- ^mice; five out of nine (56%) of the sham chimeras developed disease with a low severity of 1.2 ± 0.4. The u-PA^-/- ^→ C57 chimeras developed disease similar to u-PA^-/- ^mice (Figure 1b); 56% (seven out of 11) of the chimeras developed arthritis with a mean maximum clinical score of 1.2 ± 0.4. The severity of arthritis was significantly milder in these u-PA^-/- ^→C57 chimeras compared with C57 mice (*P *< 0.01). u-PA produced by a non-bone cell(s) is thus not sufficient for full induction of CIA.

#### Histologic features of arthritis are similar in CIA-susceptible chimeric mice as in C57BL/6 mice

**Figure 2:**
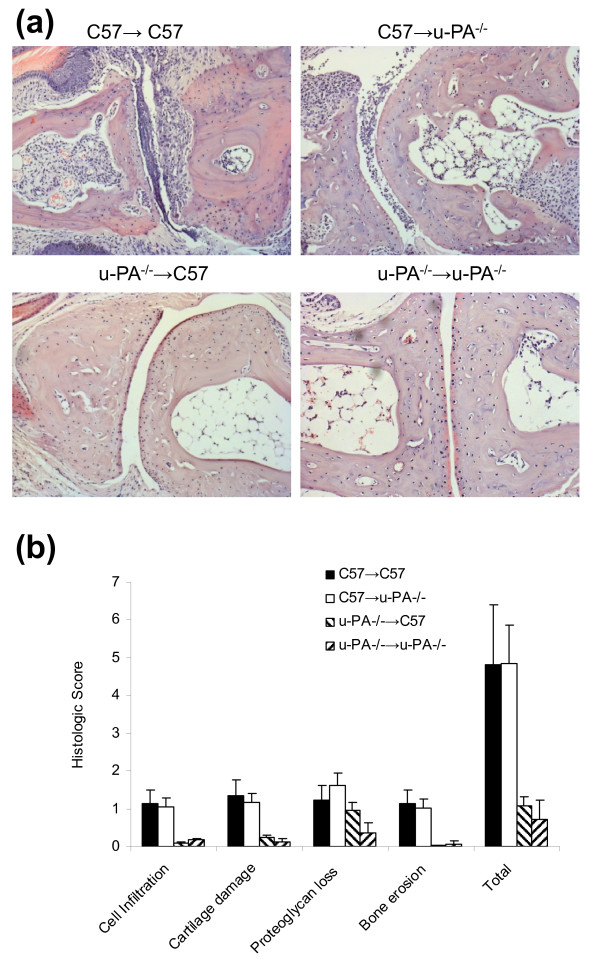
**Histologic features of arthritis are similar in arthritis-susceptible chimeric and C57BL/6 mice**. **(a) **H & E staining. Magnifications ×100. **(b) **Quantification of histologic features. C57 → C57 (n = 6 limbs), C57 → u-PA^-/- ^(n = 10 limbs), u-PA^-/- ^→ C57 (n = 6 limbs), u-PA^-/- ^→ u-PA^-/- ^(n = 6 limbs). Values expressed as mean ± standard error of the mean.

By histology, the CIA in C57 → C57 chimeras (Figure 2) and C57BL/6 mice (data not shown) [14,25,26] were indistinguishable in terms of cell infiltration, cartilage destruction, proteoglycan depletion and bone erosions. Likewise, the C57 → u-PA^-/- ^chimeras, which were susceptible to arthritis, were similar in terms of the histologic features to the C57 → C57 chimeras (Figure 2). In contrast, u-PA^-/- ^→ C57 chimeras, which developed significantly milder disease compared with C57BL/6 mice, showed minimal histologic changes which were similar to those observed in u-PA^-/- ^→ u-PA^-/- ^mice (Figure 2).

#### Inflammatory mediator production and gene expression in joints are increased in CIA-susceptible C57BL/6 mice compared with CIA-resistant u-PA^-/- ^mice

**Figure 3:**
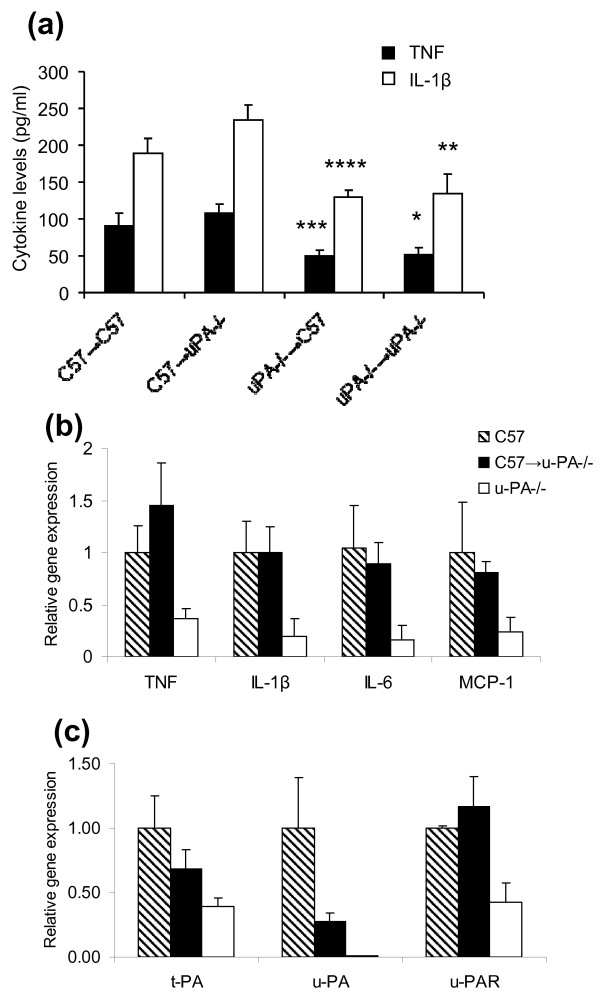
**Mediator production and gene expression in joints from mice immunized for collagen-induced arthritis**. **(a) **TNF and IL-1β levels in joint washouts following collagen-induced arthritis (CIA). TNF and IL-1β were measured by ELISA at sacrifice in washouts from ankle joints of C57 → C57 (n = 12), C57 → u-PA^-/- ^(n = 16), u-PA^-/- ^→ C57 (n = 11) and u-PA^-/- ^→ u-PA^-/- ^(n = 9) mice. *P < 0.05, **P < 0.01, C57 → u-PA^-/- ^vs. u-PA^-/- ^→ u-PA^-/- ^mice; ***P < 0.05, ****P < 0.01, u-PA^-/- ^→ C57 vs. C57 → C57 mice. **(b) **TNF, IL-1β, IL-6 and MCP-1 gene expression levels in joints following CIA (n = 6 for each group). C57 or C57 → u-PA^-/- ^vs. u-PA^-/- ^mice, *P *< 0.05 for each cytokine. **(c) **Tissue-type plasminogen activator (t-PA), urokinase-type plasminogen activator (u-PA) and urokinase-type plasminogen activator receptor (u-PAR) gene expression levels in joints following CIA (n = 6 for each group). C57 vs. u-PA^-/- ^mice, *P *< 0.05, t-PA and *P *< 0.01, u-PAR; C57 → u-PA^-/- ^vs. u-PA^-/- ^mice, *P *< 0.05, u-PAR. For (b) and (c), expression levels were normalized to an endogenous control (TATA-binding protein (TBP)) and calibrated relative to expression in C57BL/6 mice. For all, values expressed as mean level ± standard error of the mean.

Our earlier study showed that TNF and IL-1β levels were increased in joint washouts from CIA-susceptible wild-type mice compared with CIA-resistant u-PA^-/- ^mice [14]. TNF and IL-1β levels in joint washouts were significantly higher in the arthritic C57 → C57 and C57 → u-PA^-/- ^mice compared with u-PA^-/- ^→ u-PA^-/- ^and u-PA^-/- ^→ C57 mice (Figure 3a). Likewise, gene expression of TNF and IL-1β, as well as of IL-6 and MCP-1, in joints was increased in arthritic C57BL/6 and C57 → u-PA^-/- ^chimeric mice compared with u-PA^-/- ^mice following CIA development (*P *< 0.05 for each mediator, Figure 3b). Gene expression levels of joint t-PA and u-PA were also increased in arthritic C57BL/6 mice compared with u-PA^-/- ^mice (Figure 3c); however, mRNA levels in joints from arthritic C57 → u-PA^-/- ^chimeric mice were intermediate between the increased levels in arthritic C57BL/6 mice and levels in non-arthritic u-PA^-/- ^mice (Figure 3c). For u-PA, this could be explained by the fact that u-PA was only expressed in bone marrow-derived cells in C57 → u-PA^-/- ^chimeric mice. u-PAR gene expression was increased in both arthritic C57BL/6 and C57 → u-PA^-/- ^chimeric mice compared with u-PA^-/- ^mice (*P *< 0.01, C57 vs. u-PA^-/- ^mice; *P *< 0.05, C57 → u-PA^-/- ^vs. u-PA^-/- ^mice) (Figure 3c), despite the difference in u-PA expression.

**Figure 4:**
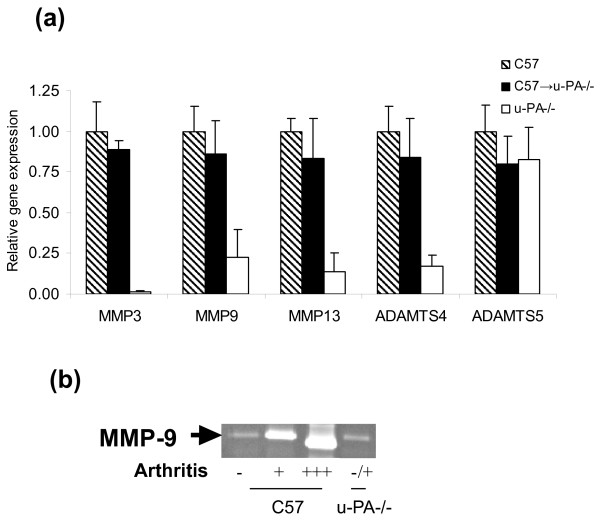
**Matrix metalloprotease and aggrecanase gene expression and MMP-9 activity in mice immunized for collagen-induced arthritis**. **(a) **Matrix metalloprotease (MMP)-3, MMP-9, MMP-13, ADAMTS-4 and ADAMTS-5 gene expression levels in joints following collagen-induced arthritis (CIA) (n = 6 for each group). C57 → u-PA^-/- ^mice, *P *< 0.01, MMP-9; *P *< 0.001, MMP-3, ADAMTS-4; and *P *< 0.0001, MMP-13. C57 → u-PA^-/- ^vs. u-PA^-/- ^mice, *P *< 0.05, MMP-9, MMP-13, and ADAMTS-4; *P *< 0.0001, MMP-3. Expression levels were normalized to an endogenous control (TATA-binding protein (TBP)) and calibrated relative to expression in C57BL/6 mice. Values expressed as mean level ± standard error of the mean. **(b) **MMP-9 levels in joint washouts, measured by zymography, from C57BL/6 and u-PA^-/- ^mice with no arthritis (-), mild arthritis (+) or severe arthritis (+++). Joint washouts were pooled (n = 3).

MMP-3, MMP-9 and MMP-13 mRNA expression levels were all increased in joints from arthritic C57BL/6 mice (*P *< 0.001, MMP-3; *P *< 0.01, MMP-9; *P *< 0.0001, MMP-13) and C57 → u-PA^-/- ^chimeric mice (*P *< 0.0001, MMP-3; *P *< 0.05, MMP-9; *P *< 0.05, MMP-13) compared with those in u-PA^-/- ^mice (Figure 4a). By zymography, MMP-9 activity was increased in joint washouts from arthritic C57BL/6 mice with increasing arthritis severity (Figure 4b). This activity in joint washouts from u-PA^-/- ^mice was similar to that in C57BL/6 mice with no arthritis, in line with these mice having no to very mild disease (Figure 4b). ADAMTS-4 mRNA expression levels, but not those for ADAMTS-5, were increased in both arthritic C57BL/6 mice (*P *< 0.001, ADAMTS-4) and C57 → u-PA^-/- ^chimeric mice (*P *< 0.05, ADAMTS-4) compared with those in u-PA^-/- ^mice (Figure 4a).

The arthritis that develops in C57 → u-PA^-/- ^chimeric mice is thus indistinguishable from the arthritis seen in C57BL/6 mice, both in terms of histologic features of disease, as well as the protein and gene expression of important inflammatory and destructive mediators.

### u-PA^-/- ^mice are resistant to type II collagen mAb-induced arthritis

We previously found [14] a reduced proliferative T-cell response to CII stimulation *in vitro *in CII-primed u-PA^-/- ^mice compared with CII-primed C57BL/6 mice even though the antibody response to CII was similar between the strains, suggesting normal immune complex formation in u-PA^-/- ^mice following CIA induction. In order to determine whether the reduced antigen-specific T-cell response could explain the mild CIA development in u-PA^-/- ^mice, the immune complex-mediated CAIA model [27] was initiated in u-PA^-/- ^and C57BL/6 mice. In contrast to CIA, this model bypasses the need for the induction of a T-cell response but requires LPS as a secondary stimulus to increase disease severity [16]. The TLR2 ligand, Pam-3-Cys, was used as a secondary stimulus, in place of LPS, as it gave more severe arthritis in C57BL/6 mice (average maximum clinical score 5. 3 ± 1.9 vs. 2.1 ± 1.3, Pam-3-Cys vs. LPS, *P *< 0.05).

u-PA^-/- ^mice were resistant to CAIA compared with C57BL/6 mice: 43% (three out of seven) of u-PA^-/- ^mice developed arthritis with an average maximum clinical score of 0.6 ± 0.2, compared with 80% (eight out of 10) of C57BL/6 mice with an average maximum clinical score of 5.3 ± 1.9 (*P *< 0.05). In fact, the three u-PA^-/- ^mice developed very mild arthritis, each with a maximum score of only 1.

### u-PA^-/- ^mice are resistant to K/BxN serum transfer arthritis

**Figure 5:**
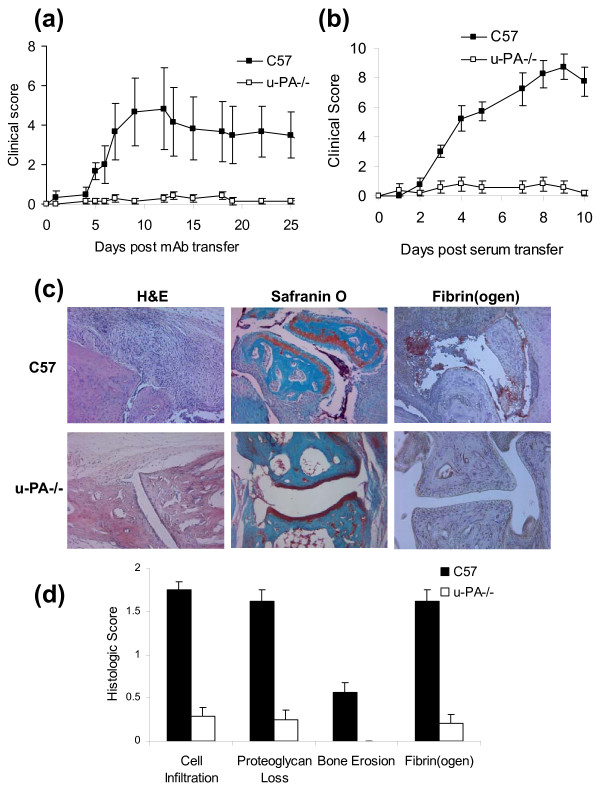
**Urokinase-type plasminogen activator gene-deficient mice are resistant to T-cell-independent arthritis models**. **(a) **Type II collagen mAb-induced arthritis (CAIA) using Pam-3-Cys as a boost. C57 (n = 10), u-PA^-/- ^(n = 7) mice. *P *< 0.05, u-PA^-/- ^vs. C57 mice. **(b) **K/BxN serum transfer model. C57 (n = 7), u-PA^-/- ^(n = 5) mice. *P *< 0.0001, u-PA^-/- ^vs. C57 mice. **(c) **Histologic pictures of representative joints from C57BL/6 and u-PA^-/- ^mice with K/BxN serum transfer arthritis. H & E, Safranin O fast green and fibrin(ogen) staining is shown. **(d) **Quantification of histologic features. *P *< 0.05, u-PA^-/- ^vs. C57 mice for each histologic feature. Values expressed as mean ± standard error of the mean.

Another immune complex-driven arthritis model is the K/BxN serum transfer model, in which serum from K/BxN mice, which develop spontaneous arthritis, is able to transfer the disease to naïve mice [19]. The arthritis that develops is more severe than the CAIA model and does not require an additional stimulus. C57BL/6 mice developed rapid and severe arthritis following serum transfer, whereas u-PA^-/- ^mice were essentially resistant (Figure 5b): 60% (three out of five) of u-PA^-/- ^mice developed arthritis with an average maximum clinical score of 1.2 ± 0.5, compared with 100% (seven out of seven) of C57BL/6 mice with an average maximum clinical score of 11.1 ± 1.3 (*P *< 0.005). Once again the three u-PA^-/- ^mice developed very mild arthritis, with a maximum score of only 2.

By histology, C57BL/6 mice show massive cellular infiltration, cartilage damage, proteoglycan loss, bone erosion and fibrin(ogen) staining following K/BxN serum transfer (Figure 5c, d). The joints from u-PA^-/- ^mice, on the other hand, look relatively normal, with minimal changes in these parameters (Figure 5c, d).

### u-PA is important for the development of immune complex-mediated peritonitis

As already mentioned, the CIA model, the CAIA model and the K/BxN serum transfer arthritis model all involve immune complex formation and deposition in joints, and, as shown above, all depend on the presence of u-PA for disease progression. From the literature there is a report showing a requirement for u-PA in immune complex-driven lung inflammation [28]. In order to explore further the proposed involvement of u-PA in immune complex-dependent inflammation we required a model where we could assess u-PA dependence following direct application of an immune complex. As the immune-complex arthritis model is induced using an intra-articular injection [29], we decided against this as such an injection leads to more severe arthritis in u-PA^-/- ^mice [12,13], possibly due to the trauma involved. For this reason, and also because the peritoneal cavity is a convenient site for the isolation and quantification of both extravasated inflammatory cells and also of inflammatory mediators, we used the immune complex-mediated peritonitis model [20].

We have previously shown that u-PA was not required for the development of murine peritonitis, as measured by neutrophil and macrophage infiltration, using either the nonspecific irritant, thioglycolate, as a stimulus, or an antigen (methylated BSA)-specific stimulus [30]. The lack of effect of u-PA deficiency with these stimuli therefore gave us the opportunity to once again see whether there is a particular association between immune complexes and u-PA.

**Figure 6:**
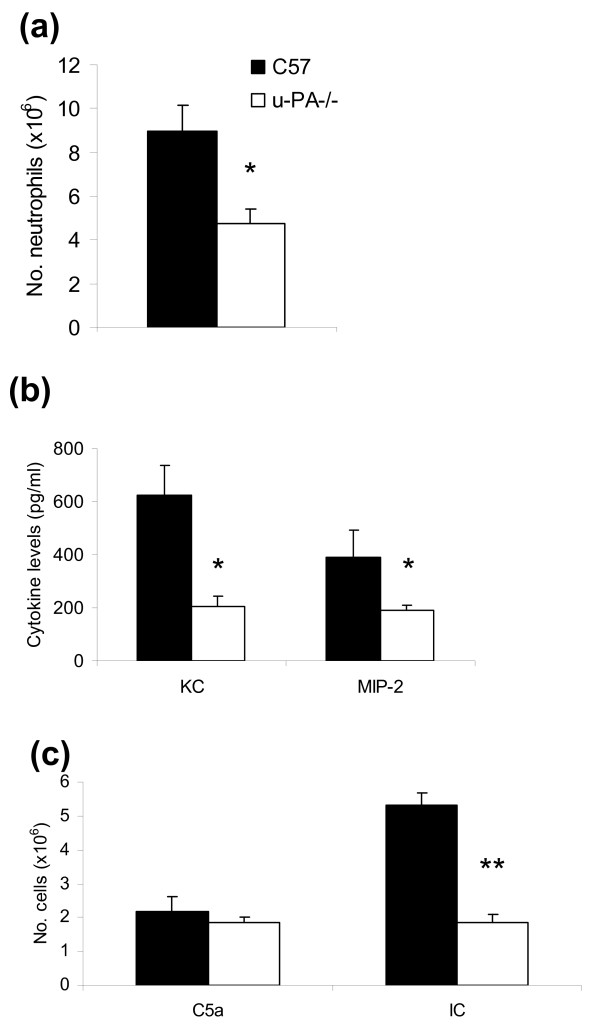
**Urokinase-type plasminogen activator is important for the development of immune complex-mediated, but not C5a-mediated, neutrophilia**. **(a) **Number of neutrophils in the peritoneal exudate of C57BL/6 (n = 12) and u-PA^-/- ^(n = 12) mice 4 hours post injection of ovalbumin (OVA) intravenously and anti-OVA intraperitoneally. **(b) **Levels of KC and MIP-2 in peritoneal exudate fluid of C57BL6 (n = 8) and u-PA^-/- ^mice (n = 8) 4 hours post OVA/anti-OVA injection, as measured by ELISA. **(c) **Total cell number in the peritoneal exudate 2 hours post intraperitoneal injection of C5a or immune complex (n = 6 per group). Values expressed as mean ± standard error of the mean. *P < 0.01, **P < 0.001, C57 vs. u-PA^-/- ^mice.

In u-PA^-/- ^mice there were significantly fewer neutrophils present in their peritoneal cavity compared with C57BL/6 mice 4 hours post initiation of immune complex-mediated neutrophilia (*P *< 0.01) (Figure 6a). The levels of the chemokines, KC and MIP-2, shown previously to be produced following immune complex-induced neutrophilia in the peritoneal cavity [20], were measured. KC (*P *< 0.01) and MIP-2 (*P *< 0.01) (Figure 6b) levels were lower in the peritoneal exudate fluid of u-PA^-/- ^mice compared with the levels in C57BL/6 mice. This suggests that u-PA is involved in the initiation of immune complex-mediated inflammatory response in the peritoneal cavity leading to the production of chemokines and recruitment of neutrophils.

There is evidence that immune complexes induce the bioactive complement component, C5a anaphylatoxin, which, in turn, interacts with the C5aR, thus reducing the threshold for Fcγ-receptor activation and leading to the recruitment of neutrophils into the peritoneal cavity [20,31]. Also, ablation of C5aR signaling abrogates neutrophil recruitment and production of KC and MIP-2 [20]. Therefore, in order to determine whether u-PA might be required downstream of C5a signaling in acute peritonitis, C5a itself was given intraperitoneally and the inflammatory response was followed. A 2-hour time point was chosen for measurement of the neutrophilia based on published literature [32]. Following intraperitoneal injection of C5a there was no significant difference in the number of recoverable peritoneal exudate cells between u-PA^-/- ^and C57BL/6 mice (Figure 6c). As a control, once again, stimulation with the immune complex led to an increased number of recoverable peritoneal exudate cells from C57BL/6 mice, with a significantly reduced number of exudate cells recoverable from u-PA^-/- ^mice 2 hours post challenge (*P *< 0.001; Figure 6c). Injection of saline alone did not induce significant numbers of neutrophils at this time point (data not shown). Since, from the data above, C5a-triggered neutrophilia in the peritoneal cavity does not require u-PA, it suggests that the u-PA involvement in immune complex-mediated neutrophilia may not be downstream of C5a signaling.

## Discussion

We and other workers have previously shown that u-PA^-/- ^mice develop very mild CIA [14,15], whereas these same mice develop more severe AIA [12] and methylated BSA/IL-1 arthritis [13] compared with C57BL/6 mice. Both the AIA and mBSA/IL-1 models are induced using an intra-articular injection resulting in monoarticular arthritis in the injected joint. In these monoarticular models, the enhanced disease severity seen in u-PA^-/- ^mice correlated with increased fibrin(ogen) deposition in the joint [12,13]. The beneficial role of u-PA in these monoarticular models is thus likely to be in fibrinolysis. The deleterious role of u-PA is less clear in CIA, with a possible effect on T-cell activation, cell migration and/or tissue destruction [1,2]. Using several polyarticular arthritis models, which all involve immune complexes and C5 activation [33,34], we showed here that u-PA is important for full disease expression.

Based on *in vitro *studies, several different cell types present in arthritic joints have been proposed to be potential sources of u-PA [10]. For CIA development, u-PA derived from a bone marrow cell(s), but not from a nonbone marrow-derived cell(s), was shown to be required for the full development of disease. A number of different bone marrow-derived cell types are required for CIA development, but lymphocytes are not required for the CAIA and K/BxN serum transfer models [35], suggesting it could be the myeloid cells that are producing u-PA required for arthritis development. Of note, the monocyte/macrophage was one cell type proposed as a source of u-PA in arthritis [10]. Gene expression of several major proinflammatory and destructive mediators were similar in the joints of CIA-susceptible chimeric mice and wild-type mice, but increased compared with CIA-resistant u-PA^-/- ^mice. Activation of procollagenases is a key control point in cartilage collagen breakdown, and it has been shown, at least *in vitro*, that the PA/plasmin system can activate MMPs [36].

It has previously been reported that u-PA was required for T-cell proliferation and activation *in vitro*, and that its absence led to a reduced T-helper type-1-polarized profile of cytokines [37]. We found T cells from CII-primed u-PA^-/- ^mice had a reduced proliferative response to CII *in vitro *compared with wild-type mice although the *in vivo *antibody response to CII was no different in u-PA^-/- ^mice, suggesting normal immune complex formation [14]. Here we found u-PA^-/- ^mice to also be resistant to the CAIA and K/BxN serum transfer models, which are T-cell-independent models, at least for disease induction [16], suggesting the defect in the antigen-specific T-cell response is unlikely to be solely responsible for the resistance of u-PA^-/- ^mice to CIA [14]. In these models, where u-PA is deleterious, its role may be in migration of leukocytes to the joints. The u-PA/u-PAR system has been implicated in migration/chemotaxis of inflammatory cells [38-43], although when using either thioglycolate or mBSA as the stimulus we found no difference in the inflammatory cell influx into the peritoneal cavity of u-PA^-/- ^mice compared with C57BL/6 mice [30], suggesting that u-PA is not always required for cell migration into tissues.

Another possible role for u-PA may be in the development of immune complex-mediated inflammation. The CIA, CAIA and the K/BxN serum transfer models all involve immune complex formation and deposition in joints, and u-PA has been reported to be required in an immune complex-induced lung inflammation model [28]. Whilst immune complexes have been reported in AIA [44], this model does not require B cells [45], and thus, disease development is not dependent on immune complex formation. Utilizing the peritoneal cavity once again we showed here that u-PA does appear to be required for immune complex-mediated neutrophilia. Immune complexes have been shown to activate the complement system leading to the generation of C5a in this peritonitis model, which then initiates the inflammatory cascade - both through direct C5aR-mediated effector functions on infiltrating and resident cells, and, also indirectly, by shifting the balance between activating (FcγRI and FcγRIII) and inhibitory (FcγRIIB) Fcγ receptors on resident cells toward an inflammatory phenotype [20]; also, ablation of C5aR signaling abrogates neutrophil recruitment and production of KC and MIP-2 in the same model [20]. By analogy, the proposed setting of a threshold for Fcγ-receptor activation in immune complex-mediated disease by C5a [20] could be occurring in the CIA, CAIA and K/BxN arthritis models, which are all C5a dependent [33,34].

As regards possible u-PA involvement in immune complex-induced lung inflammation [28], it was proposed that u-PA/u-PAR activation was necessary for C5aR signaling in alveolar macrophages, which, in turn, modulated the functional balance of the Fcγ receptors. Also, the presence of u-PA was shown to increase C5a-induced MIP-2 and TNFα production by the alveolar macrophage MH-S cell line *in vitro*, and blockade of u-PAR on the cell surface completely prevented u-PA-induced enhancement of MIP-2 and TNFα release from C5a-stimulated MH-S cells [28]. Based on these findings it was suggested that u-PA/u-PAR activation is important for effective C5a/C5aR signaling in this model, and perhaps others. We found following direct C5a administration, however, that u-PA is not required for neutrophil migration into the peritoneal cavity, suggesting that u-PA may rather be important for the generation of adequate C5a for activation/signaling downstream of immune complex activation rather than for C5a signaling itself in immune complex-driven peritonitis. In the absence of u-PA, lower levels of the chemokines KC and MIP-2 were noted in the immune complex-driven peritonitis model; it is therefore possible that there is reduced chemotaxis of neutrophils rather than there being an intrinsic defect in the ability of the cells to migrate *per se*.

Apart from signaling via its receptor, u-PA also cleaves plasminogen to form plasmin [1], which can activate complement [46]. Plasminogen^-/- ^mice have been shown to be resistant to both CIA and CAIA [15]. The relative contribution of u-PA signaling via its receptor and/or via the generation of plasmin in immune complex-mediated inflammatory responses is currently being examined as it may be model specific.

## Conclusions

u-PA is required for the full development of arthritis models involving immune complex formation and deposition - namely, the CIA, CAIA and K/BxN serum transfer models - as opposed to the monoarticular models - AIA and mBSA/IL-1. The cellular source of u-PA required for arthritis development, at least for the CIA model, is bone marrow derived and possibly of myeloid origin. From studies utilizing the peritoneal cavity and from the work of others in the lung [28], it appears that u-PA is important in other immune complex-mediated inflammatory reactions. As mentioned, any connection to complement involvement and associated mechanisms require further analysis. Given that a number of autoimmune diseases are immune complex mediated, u-PA may prove a suitable therapeutic target for such diseases.

## Abbreviations

AIA: antigen-induced arthritis; BSA: bovine serum albumin; CAIA: type II collagen mAb-induced arthritis; CII: type II collagen; CIA: collagen-induced arthritis; DMEM: Dulbecco's modified Eagle's medium; ELISA: enzyme-linked immunosorbent assay; H & E: hematoxylin and eosin; IL: interleukin; KC: Keratinocyte-derived chemokine; LPS: lipopolysaccharide; mAb: monoclonal antibody; MIP-2: macrophage inflammatory protein-2; MMP: matrix metalloprotease; PA: plasminogen activator; PBS: phosphate-buffered saline; PCR: polymerase chain reaction; t-PA: tissue-type plasminogen activator; TNF: tumor necrosis factor; u-PA: urokinase-type plasminogen activator; u-PA^-/-^: urokinase-type plasminogen activator gene deficient; u-PAR: urokinase-type plasminogen activator receptor.

## Competing interests

The authors declare that they have no competing interests.

## Authors' contributions

ADC conceived the study, and participated in its design and coordination and drafted the manuscript. CMDN carried out the K/BxN serum transfer model and immune complex-mediated peritonitis studies. ELB and ALT carried out the chimera studies. RV carried out the zymography. KJW and SKB carried out the gene and protein studies. JCL participated in the immunohistochemistry. JAH conceived the study, and participated in its design and helped to draft the manuscript. All authors read and approved the final manuscript.
